# The effect of different soft-tissue management techniques for alveolar ridge preservation: a randomized controlled clinical trial

**DOI:** 10.1186/s40729-021-00390-3

**Published:** 2021-11-19

**Authors:** Colline Papace, Christopher Büsch, Oliver Ristow, Martin Keweloh, Jürgen Hoffmann, Christian Mertens

**Affiliations:** 1 Department of Oral- and Cranio-Maxillofacial Surgery, Heidelberg University Hospital, Im Neuenheimer Feld 400, 69120 Heidelberg, Germany; 2 Private Practice, Mutlangen, Germany; 3 Institute of Medical Biometry, University of Heidelberg, Heidelberg, Germany

**Keywords:** Alveolar ridge preservation, Soft-tissue management, Tooth extraction, Surgical procedure, Randomised controlled clinical trial

## Abstract

**Purpose:**

For alveolar ridge preservation, various treatment protocols have been described. While most studies focus on the effect of the bone graft material, the aim of this study was to analyze the influence of different soft-tissue management techniques on the soft and hard tissue.

**Methods:**

A total of 20 maxillary extraction sockets were grafted with an anorganic xenogenic bone graft and then randomly treated with either a combined epithelialized-subepithelial connective tissue graft (CECG) or a porcine collagen matrix (CM) placed in labial and palatal tunnels. Measurements of soft-tissue thickness were performed at tooth extraction (T0), implant insertion (T1) and second stage surgery (T2).

**Results:**

In the CECG group, gingival thickness was 1.18 ± 0.56 mm (T0), 1.29 ± 0.26 mm (T1) and 1.2 ± 0.32 mm (T3). In the CM group, the measurements were 1.24 ± 0.50 mm (T0), 1.6 ± 0.6 mm (T1) and 1.7 ± 1.06 mm. Thus, there was an overall increase in gingival thickness from T0 to T2 of 0.02 ± 0.66 mm (CECG) compared to 0.46 ± 0.89 mm (CM). The thickness of keratinized soft-tissue was 3.91 ± 1.11 mm (CECG) and 4.76 ± 1.48 mm (CM) before extraction and 3.93 ± 1.17 mm (CECG) and 4.22 mm ± 1.26 mm (CM) at implant follow-up. Mean peri-implant probing depths were 3.15 ± 1.39 mm (CECG) and 3.41 ± 0.99 mm (CM).

**Conclusions:**

After ridge preservation, comparable soft-tissue parameters were observed in both groups, whether treated with a collagen matrix or a combined autologous connective tissue graft.

**Supplementary Information:**

The online version contains supplementary material available at 10.1186/s40729-021-00390-3.

## Introduction

Numerous clinical and preclinical studies have shown that bone remodeling after tooth extraction leads to resorption of the alveolar ridge [1, 2]. Alveolar ridge preservation is an effective technique for reducing this bone resorption if subsequent implant placement is planned in the region of the tooth extraction. In a recent systematic review and meta-analysis of alveolar ridge preservation, only randomized controlled clinical trials were analyzed [3]. The authors included 25 studies in the qualitative analysis, of which 16 studies were further processed for the meta-analysis. They showed that alveolar ridge preservation was effective in minimizing the dimensional reductions compared to an empty alveolar socket. The use of bone substitute materials resulted in a reduction in horizontal bone resorption of 1.99 mm (range 1.54–2.44 mm) and a reduced vertical resorption of 1.72 mm (range 0.96–2.48 mm). Furthermore, the authors describe the buccal bone thickness as a factor influencing the outcome. They also named other potentially influential factors, such as bone defects of the buccal wall, type of extraction site (i.e., single- vs. multi-rooted sites), or history of periodontitis, but were unable to provide proof of an influence using their existing data.

The influence of a covering soft-tissue graft, however, is rarely the main focus of studies. Vanhoutte et al. analyzed alveolar ridge preservation, where a xenogenic anorganic bone graft was covered with a connective tissue graft that was inserted into a buccal and palatal pouch [4]. They documented a stable soft-tissue contour after performing the described technique. Stimmelmayr et al. described a technique of a combined epithelized-subepithelial connective tissue graft to achieve primary wound closure of the extraction socket and, furthermore, aimed to increase the soft-tissue thickness [5, 6]. This connective-tissue graft had an epithelized central portion of the size of the extraction socket. While the connective tissue portion was inserted into the buccal and palatal pouches, the epithelized portion was the only part that was exposed to the oral cavity. In their study on 39 patients, the authors focused on the hard tissue stability of the grafted areas, not on the soft-tissue [6].

On the other hand, there have been other reports of closure of the extraction socket with lower morbidity [7] and shorter surgery time with the use of a collagen matrix instead of an autogenous soft-tissue graft [8]. A recent study also described a positive effect on the soft tissue when a collagen matrix was used.

Hence, the aim of this randomized controlled clinical study was to investigate whether the use of a porcine collagen matrix (CM) or a combined epithelialized-subepithelial connective tissue graft (CECG) had similar soft-tissue outcomes. As the combined autogenous grafts are thicker, we hypothesized that these grafts would generate a higher soft-tissue volume than the collagen matrix.

## Materials and methods

### Study design

This study was designed as a prospective, randomized, controlled, clinical study carried out according to the Declaration of Helsinki of 1975, as revised in 2000. The study protocol was approved by the ethics committees of the University of Heidelberg (application S-117/2015 of 27.04.2015) and the Baden-Württemberg Medical Association (application B-F-2015-054-z of 29.09.2015) before the start of the study. The patients were fully informed about the study, agreed to the protocol and participation was voluntary. All patients provided signed informed consent. The study was reported according to CONSORT guidelines (http://www.consort-statement.org).

### Inclusion criteria

- 18 years of age or older.
- Gingival thickness before tooth extraction > 0.8 mm.
- Fresh extraction socket without acute inflammation.
- Tooth removal in the maxilla, position 15–25.
- Intact extraction socket (buccal bone defect less than 20%).
- Planned fixed implant retained restoration.

### Exclusion criteria

- Smokers of > 10 cigarettes per day.
- Alcohol or drug abuse.
- Poor oral hygiene or compliance.
- Uncontrolled diabetes mellitus.
- Immunosuppression (chemotherapy).
- Previous or current tumor treatment.
- Pregnant or nursing mothers.
- Multi-allergic patients.
- Patients that do not adhere sufficiently to the follow-up protocol (e.g., smoking, follow-up appointments, etc.).

### Interventions

Twenty extraction sockets were randomly assigned to intervention A (CECG group) or intervention B (CM group).

The extraction sockets in both groups were first filled up to the bony margin with a bone graft substitute (Bio-Oss^®^ Collagen; Geistlich Pharma AG, Wolhusen, Switzerland). In the CECG group, the extraction socket was covered with an autologous combined connective tissue-mucosal graft from the palate, and in the CM group with a xenogenic collagen matrix (Mucograft^®^; Geistlich Pharma AG).

#### Preoperative phase

After reviewing the patients’ medical history, the clinical and radiographic findings were evaluated. Based on the PSI index, a periodontal overview was achieved by probing the sulcus using a periodontal probe and checking the bleeding tendency. This was supported by the BOP (Bleeding on Probing) findings, which were used to assess the inflammatory status of the gingiva.

#### Tooth removal (T0)

The same surgeon carried out all tooth extractions and ridge preservation procedures, implant insertions, and second stage surgeries.

The teeth were removed atraumatically using periotomes and luxators to minimize the surgical trauma, and to preserve the buccal bone and the mucosal structures. No mucoperiosteal flap was raised.

The fresh intact extraction sockets were carefully curetted to remove existing granulation tissue and promote bleeding into the socket. The bone conditions were probed and the sulcus de-epithelized. All extraction sockets were filled up to the bony margin with bone graft substitute (deproteinized bovine bone mineral with 10% collagen: Bio-Oss^®^ Collagen, Geistlich Pharma AG).

In the course of the ridge preservation procedure, the graft was covered with either a combined epithelialized-subepithelial connective tissue graft (CECG) from the palate or with collagen matrix (CM) (15 × 20 mm).

The method to be used for wound closure was selected using randomization envelopes drawn before the surgical procedure.

#### CECG group

The mesio-distal and vestibular-oral dimensions of the extraction sockets were measured using a periodontal probe and transferred to the palatal donor site. Harvesting of the graft was performed in regions 14–16 and 24–26 of the hard palate. First, the epithelialized part of the combined graft was outlined with a 1 mm deep incision. Next, horizontal incisions were added to the anterior and posterior to allow harvesting of the attached subepithelial parts of the graft [5, 6].

**Fig. 1:**
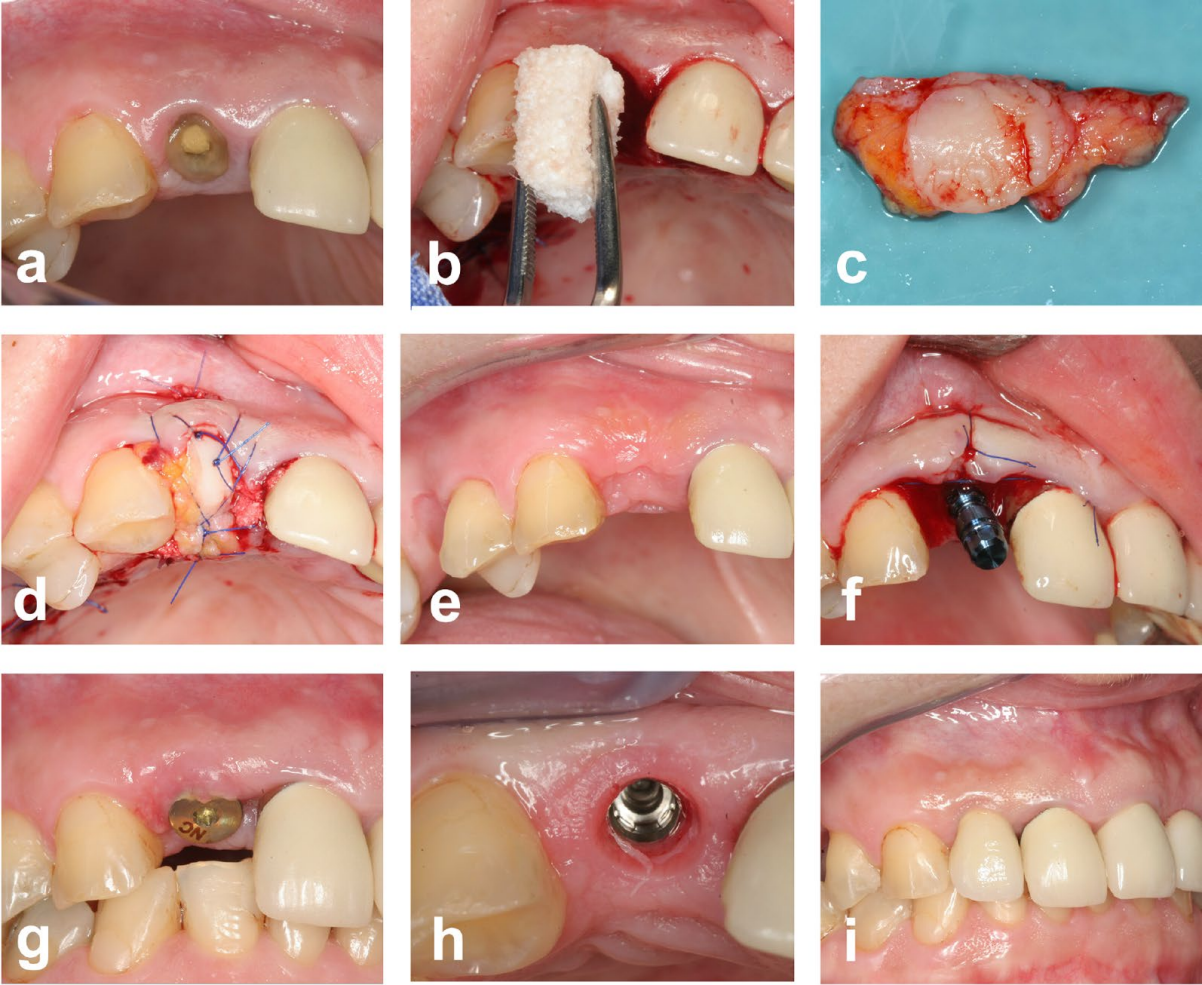
CECG group. **a** Clinical situation prior to tooth removal, **b** extraction socket is filled up to the bony margin with a xenogenic bone graft substitute, **c** autologous combined connective tissue-mucosal graft is harvested from the palate, **d** extraction socket is covered with the graft, palatal and buccal pouch, **e** clinical situation after healing, **f** implant placement without further grafting, **g** clinical situation after second stage surgery, **h** emergence profile, **i** prosthetic restoration

At the extraction socket, minimally invasive supraperiosteal tunnels were dissected, without further flap formation on the palatal and buccal sides of the socket using a scalpel and elevators to accommodate the connective tissue portions of the graft. Reverse sutures were used to insert and pull the graft into the proper position. The epithelialized part of the graft was used to seal the extraction socket. A non-absorbable monofilament suture (Dafilon 5.0) was used for suturing (Fig. 1).

#### CM group

**Fig. 2:**
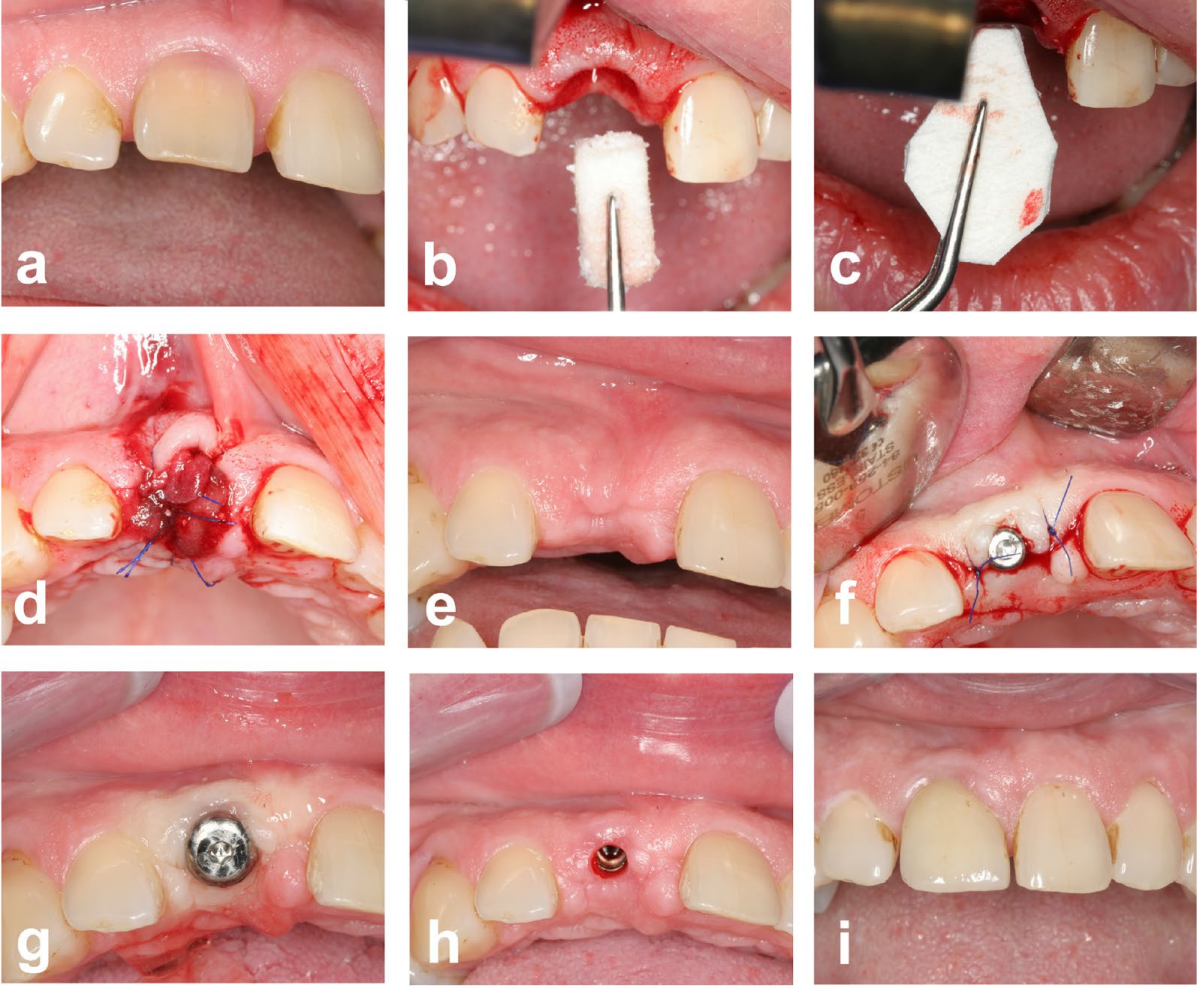
CM group. **a** Clinical situation prior to tooth removal, **b** extraction socket is filled up to the bony margin with a xenogenic bone graft substitute, **c** a xenogenic collagen matrix is adapted to fit the extraction socket and the buccal and palatal pouch, **d** extraction socket is closed with the collagen matrix, **e** clinical situation after healing, **f** implant placement without the need for further grafting, **g** clinical situation after second stage surgery, **h** emergence profile, **i** prosthetic restoration

In the intervention group, the recipient site was prepared in an identical manner and then the CM was trimmed to the defect size in a cone shape. The CM was placed and sutured buccally and palatally between the periosteum and the gingiva using reverse monofilament sutures (Fig. 2).

#### Implant insertion (T1)

Implant placement was performed after 3–4 months of healing. Prior to surgery, the thickness of the mucosa was measured, wound healing was checked and iodine solution (Lugol solution) was applied to measure the amount of keratinized mucosa. The implant lengths and diameters were determined based on cone beam computed tomography (CBCT) analyses.

A crestal incision was performed and a mucoperiosteal flap was elevated. Bone level type implants were used for this study. All implants were placed epicrestally. The implants were inserted according to the manufacturer’s instructions. The hard tissue was determined during this procedure and the necessity for secondary hard and soft-tissue augmentation was documented. Primary stability was specified and a postoperative radiograph was obtained.

#### Second stage surgery (T2)

Implants were uncovered 3–4 months after implantation. Prior to the surgery, the thickness of the mucosa was measured, wound healing was examined, Lugol solution was applied to measure the amount of keratinized mucosa, and the condition was photographically documented. Second stage surgery was performed with a slightly palatal incision and a healing abutment was inserted.

#### Follow-up (T3)

Two to three weeks after second-stage surgery, clinical examination was performed. The situation was photographically documented; radiographs were performed and the emergence profile was measured using a periodontal probe. Patients then received their fixed final restorations (7–9 months after tooth extraction).

### Outcomes

#### Primary outcome criteria

The primary outcome criterion in this study was gingival thickness. Measurements were performed after surface anesthesia (Xylocain pumpspray dental, AstraZeneca, Wedel, Germany) using a sterile root canal preparation file and an individual stent at the time of tooth extraction (T0), before implant placement (T1) and at second stage surgery (T2). After bone sounding, the thickness of the soft tissue was read off using a digital caliper gauge to measure the distance to the adjusted silicone stopper. Measurements were taken apically of the gingival margin in the middle along the tooth axis. The measurement points were located in the areas of the attached gingiva, the mucogingival line and in the mobile mucosa. The phenotype was described as thin when the measurement was 1 mm or less. In contrast, the gingival phenotype was considered thick if the measurement was more than 1 mm.

#### Secondary outcome criteria

Evaluation of the width of the keratinized gingiva was performed after staining of the mucogingival complex with Lugol stain to distinguish between attached and mobile mucosa [9]. Width was measured on the mid-buccal aspect. Over the period from tooth extraction up to second-stage surgery, the gain in keratinized gingiva was documented and determined metrically by measuring at the T0, T2 and T3 time points.

A width of the keratinized gingiva of less than 2 mm was considered insufficient, whereas a width of more than 2 mm was considered sufficient, as studies have shown less inflammation, less plaque accumulation and less recession of implants with a keratinized mucosa > 2 mm [10–12].

Measurement of the emergence profile was carried out from the implant shoulder to the crestal gingival margin using a millimeter probe.

### Sample size calculation

Statistical evaluation of the primary endpoint was confirmatory. Based on data from Cardaropoli et al. [13], case number planning was carried out for the two primary groups. For the thickening in the intervention group, an effect strength of 2.01 [13] and for the control group, an effect strength of 2.13 [13] was assumed.

With a desired power of 80% and a significance level of 5%, this resulted in *N* = 7 for the intervention group and *N* = 5 for the control group.

### Randomization

Randomization to study arms CECG and CM was performed by randomization lots with a group assignment probability of 50%. The treatment method was drawn separately for each extraction socket. The underlying randomization lots were created prior to the study, sealed in envelopes and randomly drawn by the informing physician before tooth extraction. The underlying computer-generated randomization containing the group allocation was prepared prior to the study. The randomization was conducted by the Institute of Medical Biometry according to their standardized operation procedures.

### Blinding

The same surgeon performed all therapies. To decrease the risk of bias, an observer-blind study design was chosen, hence a blinded independent observer, who was not involved in the treatment of the patients, performed the analysis.

### Statistical methods

Descriptive data were reported according to the structure and distribution of target variables, as the number of non-missing values, absolute and relative frequencies (*N*/%) for binary and categorical variables. Continuous variables were described using the number of non-missing values and mean ± standard deviation (*M* ± SD). In addition, selected target variables were pictured by the use of box plots.

As in the prospective defined study protocol, the primary outcome of “gingival thickness” between the baseline value T0 and T2 was analyzed using the Wilcoxon signed rank test for paired data for both intervention groups separately. To adjust for multiple testing, a Bonferroni adjustment for the Wilcoxon signed rank test was used, leading to a significance level of 2.5% for each of the two tests. In addition, the influence of the intervention group as well as the time on “gingival thickness” was examined using a Mann–Whitney *U* test between T2 and T0 as well as T1 and T0.

The secondary outcome criteria were analyzed using descriptive measures as well as Mann–Whitney *U* tests for group comparisons and Wilcoxon signed rank tests for time comparisons within the intervention groups separately.

All clinical assessment parameters were measured per ridge preservation, hence multiple measurements per patients exists. Missing values were not imputed. Except for the primary analysis, no formal adjustment was made for multiple testing. Hence, these *p* values can only be interpreted descriptively. *p* values smaller than 0.05 were considered to be statistically significant. Statistical analyses were conducted using the statistical software, R, version 4.0.2 (R Core Team, 2020 R Foundation for Statistical Computing, Vienna, Austria) and figures were produced using the package, ggplot2 [14].

## Results

A total of 19 patients were initially included in the study. Four patients dropped out of the study for the following reasons: two patients missed scheduled appointments. One patient terminated the study. In one patient, implant insertion was not possible, due to the presence of a residual cyst.

Finally, 15 patients with 20 extraction sockets received the intended treatment and were analyzed for the primary and secondary outcomes. Eight patients were female and seven were male. The period of recruitment was February 2016–February 2018. The last follow-up was completed in October 2018.

The patients’ selection process is shown as a CONSORT flow diagram (see Additional file 1: Fig. S1).

### Primary endpoint

#### Change in gingival thickness

**Fig. 3:**
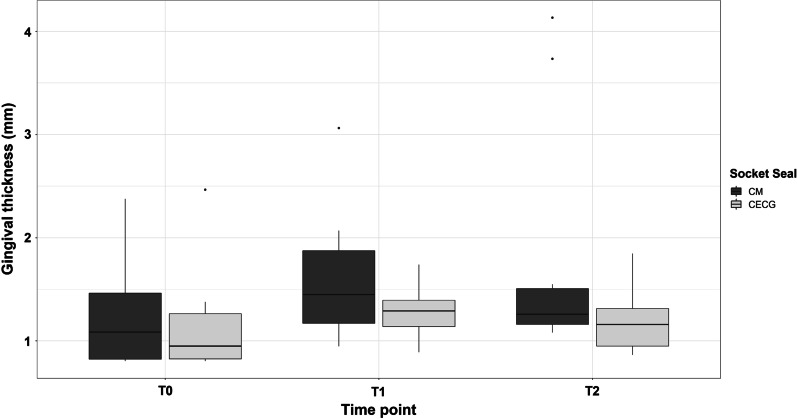
Comparison of both groups on the basis of the measured gingival thickness at the time points T0 (prior to tooth removal), T1 (prior to implant placement) and T2 (prior to second stage surgery)

Similar gingival thickness initial values of 1.18 ± 0.56 mm (CECG group) and 1.24 ± 0.50 mm (CM group) were measured at T0 in both groups. Gingival thickness had increased by the day of implant insertion (T1) to values of 1.29 ± 0.26 mm in the CECG group and 1.6 ± 0.6 mm in the CM group. At the time of second stage surgery (T2), a gingival thickness of 1.2 ± 0.32 mm was recorded in the CECG group, compared to 1.7 ± 1.06 mm in the experimental group. An overall gain in gingival thickness from T0 to T2 of 0.02 ± 0.66 mm (CECG: ${p}_{\mathrm{Wilcox}}^{\mathrm{non}-\mathrm{adj}.}$ = 0.7422, ${p}_{\mathrm{Wilcox}}^{\mathrm{adj}.}=1$) compared to 0.46 ± 0.89 mm (CM: ${p}_{\mathrm{Wilcox}}^{\mathrm{non}-\mathrm{adj}.}$ = 0.0523, ${p}_{\mathrm{Wilcox}}^{\mathrm{adj}.}=0.1045$) was achieved (Fig. 3). The recorded overall reduction difference between the two intervention groups, however, was not statistically significant (${p}_{U-\mathrm{test}}^{\mathrm{T}0-\mathrm{T}2}= 0.3544$, ${p}_{U-\mathrm{test}}^{\mathrm{T}0-\mathrm{T}1}=0.5208$).

### Secondary endpoints

#### Phenotype switching

Phenotype switching from thin to thick phenotype was observed in eight ridge preservations (CECG = four, CM = four) (Table 1).

**Table 1:** Phenotype changes per group and the phenotype over time illustrated by absolute and relative frequencies

	*N* (%)
T0 → T1	
CECG	
No change	*N* = 4/8 (50.0)
Thick → thin	*N* = 0/8 (0.0)
Thin → thick	*N* = 4/8 (50.0)
CM	
No change	*N* = 9/12 (75.0)
Thick → thin	*N* = 0/12 (0.0)
Thin → thick	*N* = 3/12 (25.0)
T1 → T2	
CECG	
No change	*N* = 6/8 (75.0)
Thick → thin	*N* = 2/8 (25.0)
Thin → thick	*N* = 0/8 (0.0)
CM	
No change	*N* = 11/12 (91.6)
Thick → thin	*N* = 0/12 (0.0)
Thin → thick	*N* = 1/12 (0.83)
T0 → T2	
CECG	
No change	*N* = 4/8 (50.0)
Thick → thin	*N* = 1/8 (12.5)
Thin → thick	*N* = 3/8 (37.5)
CM	
No change	*N* = 8/12 (66.7)
Thick → thin	*N* = 0/12 (0)
Thin → thick	*N* = 4/12 (33.3)

#### Change of keratinization

**Fig. 4:**
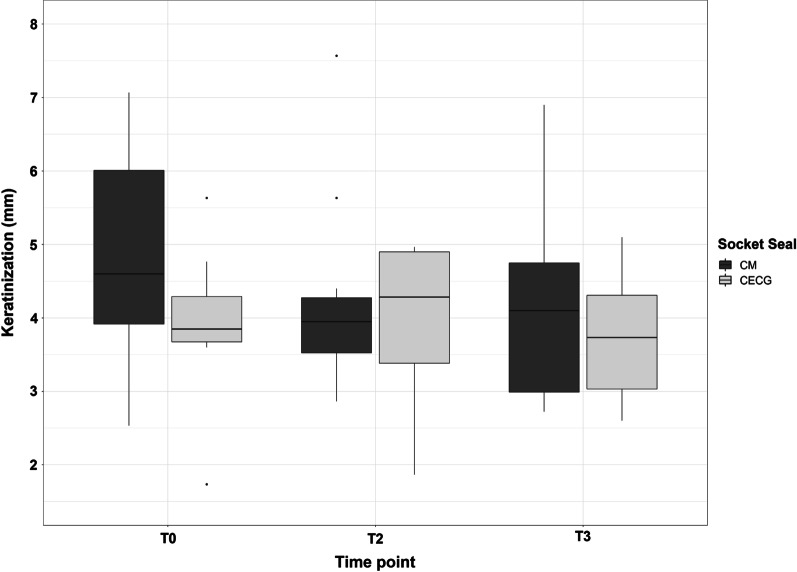
Comparison of both groups on the basis of the measured widths of the keratinized gingiva at the time points T0 (prior to tooth removal), T2 (after second stage surgery) and T3 (after prosthetic restoration)

At T0, initial keratinization of 3.91 ± 1.11 mm (CECG) and 4.76 ± 1.48 mm (CM) were measured. At T2, keratinization was 3.93 ± 1.17 mm and 4.22 ± 1.26 mm, respectively, while at T3, it was 3.73 ± 0.90 mm (CECG) and 4.13 ± 1.29 mm (CM) (Table 2  & Fig. 4).

**Table 2:** Mean values and standard deviation of the measured widths of the keratinzed gingiva at the T0 (before tooth removal), T2 (after second stage surgery) and T3 (after prosthetic restoration)

Descriptive statistics			
	*Group*	Mean	SD
T0	CECG (*N* = 8)	3.91	1.11
	CM (*N* = 12)	4.76	1.48
	Total (*N* = 20)	4.42	1.38
T2	CECG (*N* = 8)	3.93	1.17
	CM (*N* = 12)	4.22	1.26
	Total (*N* = 20)	4.10	1.20
T3	CECG (*N* = 8)	3.73	0.90
	CM (*N* = 12)	4.13	1.29
	Total (*N* = 20)	3.97	1.14

The intervention group effect in mucosal keratinization was investigated by Mann–Whitney *U* tests using the mucosal keratinization difference between T0 and T3 or T4 separately. No differences could be found between the two groups with regard to the keratinization of the gingiva (${p}_{U-\mathrm{test}}^{\mathrm{T}0-\mathrm{T}3}=0.6784$, ${p}_{U-\mathrm{test}}^{\mathrm{T}0-\mathrm{T}4}= 3746$).

#### Complete keratinization

The group differences with respect to the time of complete keratinization of the graft were calculated using the Mann–Whitney test based on the ordinal scale level. No significant difference could be found (${p}_{U-\mathrm{test}}=0.3587$) (Table 3).

**Table 3:** Complete keratinization per Socket Seal type over time illustrated by absolute and relative frequencies

Complete keratinization			
	Socket seal		Total (*N* = 20)
	CECG (*N* = 8)	CM (*N* = 12)	
Complete keratinization			
6 weeks postoperative	1	0	1
	12.5%	0.0%	5.0%
8 weeks postoperative	0	3	3
	0.0%	25.0%	15.0%
10 weeks postoperative	0	1	1
	0.0%	8.3%	5.0%
12 weeks postoperative	1	2	3
	12.5%	16.7%	15.0%
> 12 weeks postoperative	6	6	12
	75.0%	50.0%	60.0%

#### Emergence profile

**Fig. 5:**
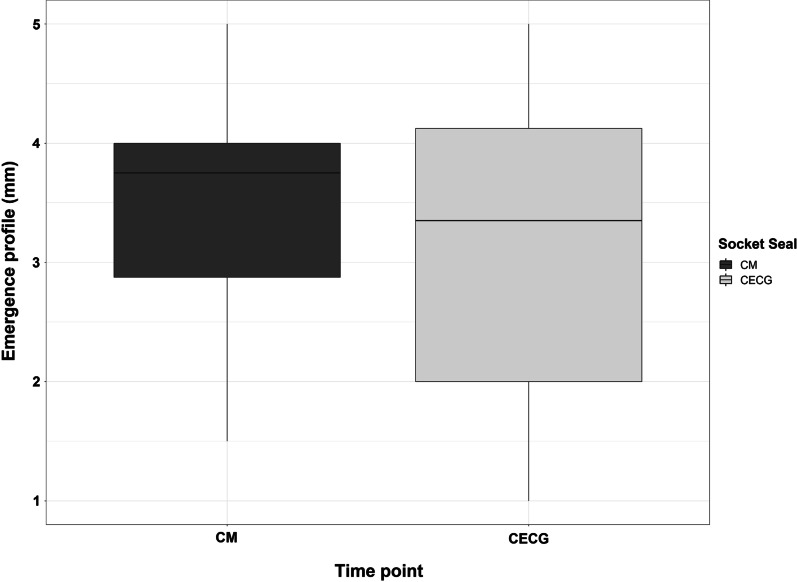
Comparison of emergence profile in both groups after second stage surgery (T2)

Measurement of the emergence profile was carried out from the implant shoulder to the crestal gingival margin using a millimeter probe. There was no significant difference between the two therapy groups: CECG group: 3.15 ± 1.39 mm; CM group: 3.41 ± 0.99 (${p}_{U-\mathrm{test}}=0.7858$) (Fig. 5).

#### Marginal bone level

All implants could be placed at the time of implant placement (T1) without the need for secondary hard or soft-tissue augmentation.

##### Mesial

At time point T1, mesial bone levels of 0.24 ± 1.11 mm and 0.70 ± 1.23 mm in the CECG and CM group, respectively, were recorded. A similar group difference of 1.36 ± 1.55 mm (CECG) and 1.07 ± 1.21 mm (CM) was measured at time point T2 (Table 4). These group differences in the mesial bone level at both timepoints (T1 and T2) were not significant (${p}_{U-\mathrm{test}}^{\mathrm{T}1 }=0.5609$, ${p}_{U-\mathrm{test}}^{\mathrm{T}2 }=0.5110$). Moreover, the mesial bone level did not change significantly over time in either intervention group (CECG: ${p}_{\mathrm{Wilcox}}=0.0797$; CM: ${p}_{\mathrm{Wilcox}}=0.2892$).

**Table 4:** Mean values and standard deviation of the measured mesial bone level at the time of implant placement (T1) and second stage surgery (T2)

Mesial		
	Mean	SD
Marginal bone T1 mesial (mm)		
CECG (*N* = 8)	0.24	1.11
CM (*N* = 12)	0.70	1.23
Total (*N* = 20)	0.52	1.18
Marginal bone T2 mesial (mm)		
CECG (*N* = 8)	1.36	1.55
CM (*N* = 12)	1.07	1.21
Total (*N* = 20)	1.19	1.33

##### Distal

At timepoint T1 a distal bone level of 0.39 ± 1.19 mm and 0.34 ± 1.18 mm in the CECG and CM group was recorded, respectively. A larger group difference of 0.24 ± 1.17 mm (CECG) and 1.03 ± 1.11 mm (CM) was measured at timepoint T2 (Table 5). These group differences, however, at both timepoints (T1 and T2) were not significant (${p}_{U-\mathrm{test}}^{\mathrm{T}1 }=0.9077$, ${p}_{U-\mathrm{test}}^{\mathrm{T}2 }=0.1640$). In addition, analysis investigating the time difference in each group separately showed that the distal bone level in the CM group increased significantly between timepoints T1 and T2 (${p}_{\mathrm{Wilcox}}=0.0161$). No significant difference was found in the CECG group (:${p}_{\mathrm{Wilcox}}=0.7422$).

**Table 5:** Mean values and standard deviation of the measured distal bone level at the time of implant placement (T1) and second stage surgery (T2)

Distal		
	Mean	SD
Marginal bone T1 distal (mm)		
CECG (*N* = 8)	0.39	1.19
CM (*N* = 12)	0.34	1.18
Total (*N* = 20)	0.36	1.15
Marginal bone T2 distal (mm)		
CECG (*N* = 8)	0.24	1.17
CM (*N* = 12)	1.03	1.11
Total (*N* = 20)	0.71	1.17

## Discussion

The aim of this study was to determine whether covering an extraction socket after alveolar ridge preservation with either a combined epithelialized-subepithelial connective tissue graft or porcine collagen matrix had an influence on the soft- and hard-tissue outcome, in particular the soft-tissue thickness.

As most studies analyzing alveolar ridge preservation procedures focus on the type of graft material used to augment the extraction socket [3], this study investigated the influence of soft-tissue management as the influencing factor. The grafting procedure of the extraction sockets prior to the soft-tissue management was identical in both groups and used the same xenogenic graft material that is well documented in the literature. To achieve comparable data, only intact extraction sockets (maximum allowed buccal bone loss of 20% visible by CBCT) of the upper maxilla from region 015–025 were included in this study.

Furthermore, the included patients were selected according to predefined inclusion and exclusion criteria to obtain a more homogenous patient group and were randomized according to the study design to one of two groups. In addition, one surgeon performed all surgical procedures and an independent observer who was not involved in the treatment of the patients performed the analysis to decrease the risk of bias.

A limitation of this study is the small sample size; however, the number of included patients is similar to other studies on the same subject and a sample size calculation was performed prior to the start of this study.

In the CECG group a combined epithelialized-subepithelial connective tissue graft was used as soft-tissue graft. According to Stimmelmayr et al., this type of graft has the advantage of thickening the mucosa and promoting local conversion of a thin marginal gingiva to a thick marginal gingiva [5, 6]. Furthermore, due to the tunneling procedure, the mucogingival line stays in place. In addition, vascularization is claimed to be more reliable due to the connective tissue pedicles that are placed in the prepared pouches.

In the experimental group, a xenogenic collagen matrix was used. Here the matrix was adapted to the same dimensions as the CECG with large parts being positioned in the prepared pouches.

In this study, a procedure without flap formation was performed, as some studies reported increased bone resorption due to flap elevation and the resulting disruption of periosteal vascularization [15]. The thin buccal bone can be 0.2–0.4 mm and is highly tooth dependent.

The primary outcome was gingival thickness, which was measured at three different timepoints. An overall change in gingival thickness from T0 to T2 of 0.02 ± 0.66 mm was recorded for the CECG group compared to 0.46 ± 0.89 mm for the CM group. Differences between the two groups were not statistically significant. These findings might indicate that CM and CECG produce similar results. To prove this hypothesis, however, further research is required.

A stable thickening of the soft tissue in the sense of phenotype switching from a thin to a thick phenotype was shown by a total of seven ridge preservations over time from T0 to T2, with almost no group difference (CM: 4 (33.3%), CECG: 3 (37.5%)). Over the whole time-span from T0 to T2, only one ridge preservation in the CECG group exhibited phenotype switching from a thick to a thin phenotype.

Regarding the emergence profile, no significant difference was found between the two therapy groups (*p* = 0.7858), with the CECG group at 3.15 ± 1.39 mm, and the CM group at 3.41 ± 0.99 mm.

Both groups showed clinical soft-tissue healing without complications within 3–4 weeks. In addition, compared with autologous CECG, CM was characterized by an equivalent and non-differentiating adaptation to the adjacent tissue, although this aspect should also be evaluated objectively in future studies. An initial keratinization in the CM group was observed after 8 weeks at the earliest.

Furthermore, preservation of the alveolar ridge can simplify the course of further treatment, because augmentation may be necessary less frequently. Furthermore, implant placement can be performed in the ideal prosthetic position. This positive effect could also be demonstrated in the current study. All implants could be placed at the time of planned implant placement (T1), without the need for secondary hard or soft-tissue augmentation.

## Conclusions

After ridge preservation using a standardized approach, comparable soft-tissue parameters were observed in both groups, either with a collagen matrix or a combined autologous connective tissue graft. As an example, there was almost no measurable difference in the overall gain of gingival thickness between the two interventions. In addition, there were no significant differences in outcomes between the two groups, indicating that the CM and CECG interventions both lead to similar results. To prove this hypothesis, however, further research is required.

## Supplementary Information

**Additional file 1.** CONSORT 2010 flow diagram.

## Data Availability

The data sets used and/or analyzed during the current study are available from the corresponding author on reasonable request.

## Abbreviations

CBCT: Cone beam computed tomography
CM: Collagen matrix
CECG: Combined epithelialized-subepithelial connective tissue graft
T0: Tooth removal
T1: Implant insertion
T2: Second stage surgery
T3: Follow-up
