# Supplementary material for: The effect of different soft-tissue management techniques for alveolar ridge preservation: a randomized controlled clinical trial
Source: Int J Implant Dent. 2021 Nov 19;7:113. doi: 10.1186/s40729-021-00390-3 (PMC8603978; doi:10.1186/s40729-021-00390-3)
Supplement: Supplementary file 1 — Additional file 1. CONSORT 2010 flow diagram. [file 40729_2021_390_MOESM1_ESM.doc]

**
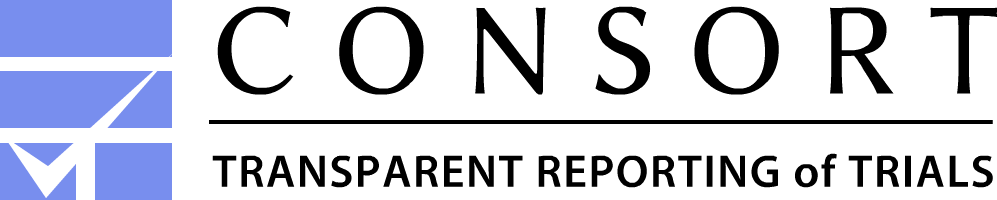
**

**CONSORT 2010 Flow Diagram**

**Allocation**

**Analysis**

**Follow-Up**

**Enrollment**

Assessed for eligibility (N=22 patients with 24 implants)

Excluded (N=0)

Analysed (N=11 patients with 12 implants)
♦ Excluded from analysis (N=0)

Lost to follow-up (N=1 patient with 1 implant)
♦ One patient terminated the study.

Discontinued intervention (N=2 patients with 2 implants)
 ♦ One patient, implant insertion was not possible, due to presence of a residual cyst.
♦ One patient missed scheduled appointments.

Allocated to intervention CM

(N= 14 patients with 15 implants)

♦ Received allocated intervention (N=12)

♦ Did not receive allocated intervention (N=3)

Lost to follow-up (N=1 patient with 1 implant)
♦ One patient missed scheduled appointments.

Allocated to intervention CECG

(N=8 patients with 9 implants)

♦ Received allocated intervention (N=8)

♦ Did not receive allocated intervention (N=1)

Analysed (N= 7 patients with 8 implants)
♦ Excluded from analysis (N=0)

Randomized (N=22 patients with 24 implants)
